# The APPEESFRS Peptide, Restricted by the HLA-B*35:01 Molecule, and the APPEESFRF Variant Derived from an Autologous HIV-1 Strain Induces Polyfunctional Responses in CD8+ T Cells

**DOI:** 10.1089/biores.2014.0054

**Published:** 2015-01-01

**Authors:** Liliana Acevedo-Sáenz, Liseth Carmona-Pérez, Paula Andrea Velilla-Hernández, Julio C. Delgado, María Teresa Rugeles L.

**Affiliations:** ^1^ Grupo Inmunovirología, Facultad de Medicina, Universidad de Antioquia (UdeA), Medellín, Colombia.; ^2^ ARUP Institute for Clinical and Experimental Pathology, Department of Pathology, University of Utah School of Medicine, Salt Lake City, Utah.

**Keywords:** human immunodeficiency virus, functional profile, peptides, HIV-specific CD8+ T-cells

## Abstract

Numerous reports have focused on consensus peptides to determine CD8+ T-cell responses; however, few studies evaluated the functional profile using peptides derived from circulating strains of a specific region. We determined the effector profile and maturation phenotype of CD8+ T-cells targeting the consensus APPEESFRS (AS9) epitope and its variant APPEESFRF (AF9), previously identified. The free energy of binding, maturation phenotype, and polyfunctional profile of both peptides were similar. The magnitude of CD8+ T-cell responses to AF9 was greater than the one elicited by AS9, although the difference was not significant. The polyfunctional profile of AF9 was characterized by CD107a/interleukin-2 (IL-2)/macrophage inflammatory protein beta (MIP1β) and by interferon gamma (IFNγ)/MIP1β/tumor necrosis factor alpha (TNFα) in response to AS9. TNFα production was significantly higher in response to AF9 than to AS9, and there was a negative correlation between the absolute number of CD8+ T-cell-producing TNFα and the plasma human immunodeficiency virus (HIV) load, suggesting a role of this cytokine in the control of HIV replication.

## Introduction

CD8+ T cells play an important role in the control of human immunodeficiency virus (HIV) replication, both in the acute and chronic phases of infection.^1,2^ Several studies have focused on the identification of immunodominant epitopes of CD8+ T cells, capable of inducing strong specific responses;^3^ however, such studies have been hampered by the high genetic variability of the virus.^4,5^ Given this genetic heterogeneity, the evaluation of HIV-specific CD8+ T cells is frequently performed using consensus peptides derived from viral sequences belonging to the M group^6^ of HIV-1. This approach is practical, has cost–benefit advantages, and has generated a large amount of information; however, few studies have focused on the evaluation of the functional profile of HIV-specific CD8+ T cells in response to peptides derived from circulating strains of a specific region (autologous peptides) that may vary between strains of the same HIV subtype.^7^ A study of Doroudchi et al. reported that a greater magnitude of CD8+ T cells expressing interferon gamma (IFNγ) was observed when the cells were stimulated with autologous instead of consensus HIV-1 peptides derived from the Nef protein, underlying the importance of using circulating strains to define immunogenic peptides with immunotherapy potential.^8^

In a previous study we identified the autologous APPEESFRF peptide (Gag 457–465), presented by the human leukocyte antigen-B*35:01 (HLA-B*35:01) molecule. This peptide has a phenylalanine (F) at position 9, while the reference sequence HXB2 carries a serine (S) in this position. Taking into consideration that a single amino acid change in the peptide could affect/eliminate the binding to the major histocompatibility complex (MHC) molecule, reducing the recognition of the peptide by the T cell receptor or inducing an inefficient CD8+ T cell response,^9–11^ our objective was to evaluate the influence of the S465F mutation in the APPEESFRS peptide on the functional profile of HLA-B*35:01-restricted CD8+ T cells, in Colombian HIV-1 chronically infected patients.

## Materials and Methods

The Gag gene was amplified from 43 DNA samples from chronic asymptomatic HIV-1-infected adults, using the polymerase chain reaction (PCR) conditions and the primers previously described.^12^ These patients were highly active antiretroviral therapy naïve, had detectable viral loads (>50 and <100,000 RNA copies/mL) and CD4+ T cell count higher than 350 cell/μL.

The products were sequenced using the Sanger method, and then edited in the SeqMan NGen® versión 3 program (DNASTAR), aligned using the ClustalW method, and translated using the Bioedit software (Ibis Bioscience) to determine the frequency of the S465F mutation.

To establish the *in silico* docking simulation assay for peptides binding to the HLA-B*35:01 molecules, the three-dimensional structure of the molecule was downloaded from the Protein Data Bank (PDB) database (www.pdb.org) (PDB code 1A1N); for the three-dimensional structural peptides, the homology-modeling ESyPred3D server^13^ was used. The affinity constant based on the indirect score of the free energy of binding was evaluated, using the receptor-ligand docking simulation software, AutodockVina.^14^

To evaluate the functional profile and memory phenotype of HIV-specific CD8+ T cells, we incubated peripheral blood mononuclear cells (PBMCs) obtained from five chronically infected HIV-1 individuals expressing HLA-B*35:01 allele with brefeldin A (Sigma-Aldrich), monensin, anti-CD28 (CD28.2), anti-CD49d (9F10) (eBioscience), and CD107a-APC (H4A3) (BD Pharmingen), and stimulated the PBMCs for 12 h with 10 μg/mL of each peptide (APPEESFRS or APPEESFRF). Cells cultured with anti-CD28 and anti-CD49d served as background, and PBMCs stimulated with *Staphylococcus* enterotoxin B were used as positive control. The cells were stained with the following antibodies: CD3 APC-Cy7 (clone UCHT1), CD8 Pacific blue (clone RPA-T8), CD127 APC (clone eBioRDR5), CD45RA PE-Cy5 (clone HI100), and CD197 (CCR7) PE (clone 3D12) (eBioscience). Next, cells were washed and fixed/permeabilized, using the Foxp3 staining buffer set (eBioscience) and stained with the following antibodies against intracellular markers: tumor necrosis factor gamma (TNFα) PerCP-Cy5.5 (clone Mab11, eBioscience), CD107a APC (clone eBioH4A3), interferon gamma (IFNγ) PeCy7 (clone 4S.B3), interleukin-2 (IL-2) FITC (clone MQ1-17H12) and macrophage inflammatory protein 1 beta (MIP1β) PE (clone D21-1351) (BD Pharmingen). Finally, the cells were washed, fixed with 1% formaldehyde, and analyzed using the FACS Canto II cytometer (BD Immunocytometry Systems). A minimum of 200,000 events were acquired per sample and the polyfunctional analyses were carried out using the Boolean gating strategy (as indicated in the FlowJo software) to generate possible combinations of the responses. In the analyses, all data were background-subtracted using the CD28/CD49d stimulation and a positive response was reported after background correction and the percentage of epitope-specific CD8+ T cell response had to be at least two times higher than background, for each tested marker. A polyfunctional CD8+ T cell response refers to three or more effector molecules induced by a given peptide. The presentation of distributions was performed using the SPICE version 5.3 (downloaded at http://exon.niaid.nih.gov/spice).^15^

The study was carried out with samples collected in a previous study, which was approved by the University of Antioquia Ethical Review Board. All participants signed an informed consent, which included an extension allowing the use of remaining samples for future studies related to HIV-1 infection. This consent was prepared according to the Colombian legislation (Resolution 008430 of 1993).

## Results and Discussion

To determinate the frequency of S465F in the Gag region, we amplified 43 sequences and observed that 100% of the sequences exhibited the change of serine (S) to phenylalanine (F). Indeed, this variant was previously reported and seems to confer an advantage to the virus, since the polymorphism is now fixed in several viral strains (HIV Molecular Immunology Database of Los Alamos HIV (www.lanl.gov/content/immunology). The APPEESFRF peptide is located between positions 9 and 17 of the p6 protein, and the last three residues of this peptide are part of the conserved domain, _15_F_16_R_17_F_18_G which interacts with the Vpr protein, promoting virion packaging; remarkably, a single amino acid change at position 17 abolishes Vpr packaging, pointing the importance of the FRFG domain in different viral strains.^16^ Considering that the S465F mutation corresponds to a nonconservative change, natural selection rather than random genetic shift is the most likely mechanism behind this variant.^17^

**FIG. 1.**
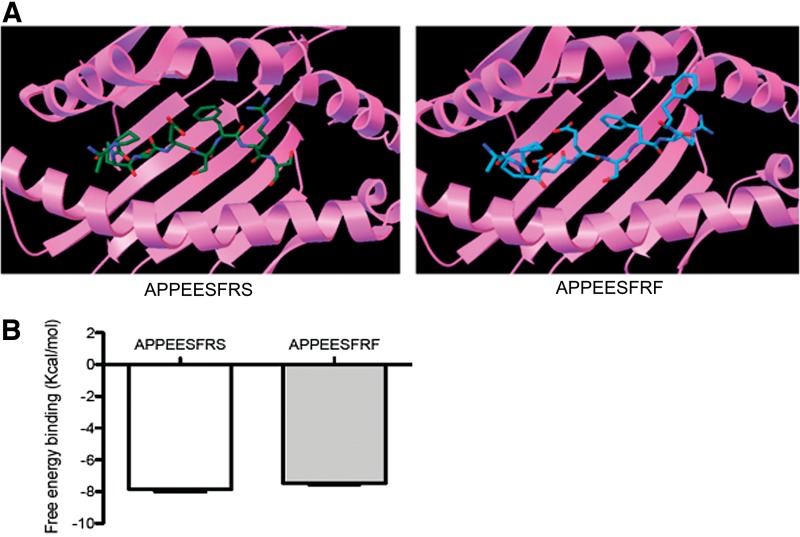
Characterization of the binding of APPEESFRS- and APPEESFRF-restricted HLA-B*35:01 peptides using the docking simulation assay. An example figure of our *in silico* docking simulation assay is shown in **(A)**. The mean and standard deviation of the 20 lowest binding free energies are shown in **(B)** and Mann-Whitney U test was used for statistical analysis.

The results of the docking simulation assay indicate that both peptides exhibit a similar free energy of binding to the HLA molecule (AS9=−7.9 and AF9 −7.45 Kcal/mol) (Fig. 1A, B), suggesting that this amino acid change does not influence the overall binding ability to the MHC molecule. Similar binding of both peptides to the HLA-B*35:01 molecule might be explained by the strong preference for the aliphatic hydrophobic residues proline (P) and alanine (A) at position 2, and tyrosine (Y) at position 9 (C-terminal) of this allele.^18^ The three dimensional structure of the HLA-B*35:01 molecule exhibits a S at residue 116 and a Y at residue 74 in the F pocket, allowing the formation of hydrogen bonds between these amino acids with hydroxyl groups.^18^ In addition, the F pocket in molecules of the B7 supertype, such as HLA-B*35:01, prefer binding to hydrophobic aliphatic (alanine, leucine, isoleucine, methionine, or valine) or to large aromatic hydrophobic residues, such as F and Y.^19^

We then evaluated the functional profile and the maturation phenotype in HLA-B*35:01-restricted CD8+ T cells stimulated with both peptides. We observed that the effector terminally phenotype (CD45RA+CCR7−CD127−) controlled the specific CD8+ T cells, followed by the effector phenotype (CD45RA−CCR7−CD127−), in response to both peptides, supporting previous studies that reported a similar memory phenotype in chronically infected HIV-1 individuals.^20,21^

**FIG. 2.**
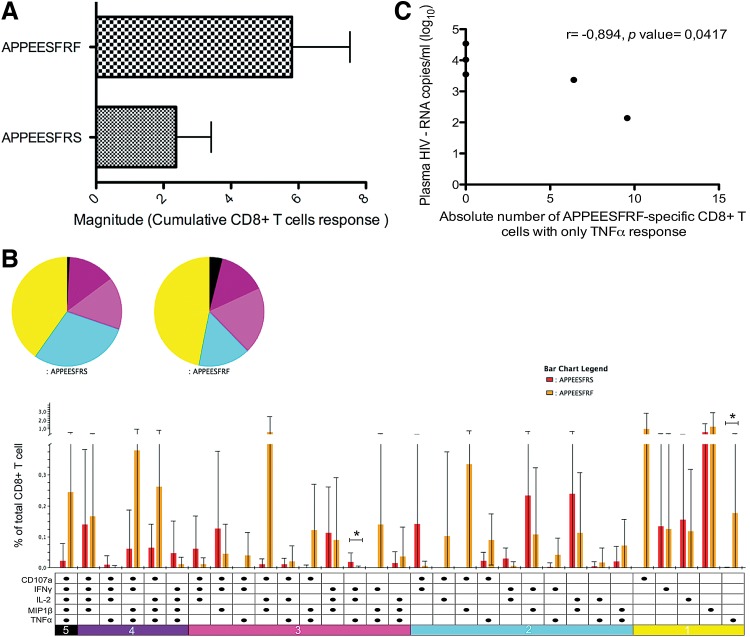
Magnitude and functional profile of APPEESFRS- and APPEESFRF-specific CD8+ T cells. Comparison of magnitudes expressed in percentage of cumulative CD8+ T cells **(A)** and comparison of the functional profiles **(B)** of APPEESFRS and APPEESFRF-specific responses; Mann-Whitney U test was used and the statistical significance was indicated by an asterisk (*p*<0.05). Plasma viral load was plotted as a function of the absolute number of APPEESFRF-specific CD8+ T cells expressing tumor necrosis factor-alpha (TNFα) **(C)**. Each dot represents each individual and Spearman *R* test was used for statistical analysis.

The total magnitude of CD8+ T cell response was greater in the autologous peptide compared to the consensus peptide (AS9, 2.370%; AF9, 5.808%); although the difference was not statistically significant (Fig. 2A).

Monofunctional cells contributed the most to the global response to both peptides (AS9, 40%; AF9, 47%), where the effector molecules that predominated for AS9 were MIP1β, IL-2, or IFNγ, whereas for AF9 they were CD107a, MIP1β or TNFα (Fig. 2B).

For both peptides, a polyfuntional response was observed. In particular for the consensus AS9, the most frequent functional response was two, followed by three, four, and five functions (29%, 16%, 14%, and 1% respectively), whereas for AF9 the most frequent response was three, followed by two, four, and five functions (20%, 15%, 14%, and 4%, respectively). Although the polyfunctional response was not confined to a particular combination of functional parameters, a trend to a higher frequency of cells producing CD107a IL-2 MIP1β or IFNγ MIP1β TNFα in response to AF9 or AS9, respectively, was observed (Fig. 2B). In general, the responses to either peptide could be associated with viral control; previous studies have indeed suggested that polyfunctional profiles are associated with slow progression to acquired immune deficiency syndrome; furthermore, the quality of the CD8^+^ T cell functional response has been proposed to serve as an immune correlate of HIV disease progression.^22^ Similarly, a previous study reported that the magnitude of Gag- and Nef-specific CD8+ T cell values were inversely correlated with the viral set point; Gag-specific CD8+ T cells with four functions was significantly higher in individuals with the lowest viral load, while in Gag-specific CD8+ T cells exhibiting two functions it was higher in individuals with the highest viral load.^23^

On the other hand, the proportion of cells producing IL-2, TNFα, and IFNγ was significantly higher in response to AS9 compared with AF9 (*p*=0.0437), and the expression of TNFα alone was significantly higher in response to AF9 compared with AS9 (*p*=0.0234). However, we only observed a negative correlation with the absolute number of AF9-specific CD8+ T cells producing TNFα alone and the plasma HIV viral load (copies/mL) (*r*=−0.894, *p*=0.0417) (Fig. 2C), suggesting an important role of this cytokine in viral control.

TNFα plays an essential role in host defense, but during HIV infection the precise role of this cytokine is not completely understood and controversial results have been published.^24^ TNFα has been associated with decreased time to reach a CD4+ T cell count <500 cells/mm^3^ and the initiation of antiretroviral therapy during early HIV-1 infection in untreated primary HIV-1 infection;^25^ in addition, TNFαhas been shown to induce HIV-1 replication in infected cells through the translocation of NF-κB to the nucleus followed by activation of the HIV long terminal repeat.^26^ In contrast, other studies have demonstrated the anti-HIV potential of this cytokine by one of the following mechanisms: (1) downregulation of HIV receptors and coreceptors at the cell surface,^27^ (2) inhibition of HIV replication in several cell types,^28^ (3) induction of RANTES, MIP1α, and MIP1β, factors with HIV suppression activity,^29^ and (4) induction of apoptosis in HIV-infected cells,^30^ which could all account for the negative correlation with HIV load.

## Conclusion

In summary, we observed a polyfunctional profile in response to consensus and autologous peptides, TNFα being the cytokine that seems to play a key role in controlling viral replication. These results suggest that both peptides could be considered in immunological therapy, although the autologous peptide seems to have a greater capacity to elicit stronger responses, as supported by previous studies, and contrast with previous studies in which the response to autologous peptides was much stronger compared to the one elicited by consensus peptides.^8,31^ However, it is necessary to take into account several limitations of this study. The stability and strength of the binding were not explored; modeling the interaction between the S9F residues and the MHC residues was not carried out.^32^ Furthermore, the number of samples used to determine the functional profile was limited, making it difficult to explore possible associations with immunological and virological parameters.

## Abbreviations Used

A: alanine
AF9: APPEESFRF
AS9: APPEESFRS
F: phenylalanine
HAART: highly active antiretroviral therapy
HIV: human immunodeficiency virus
HLA: human leukocyte antigen
IFNγ: interferon gamma
IL-2: interleukin 2
LTR: long terminal repeat
MHC: major histocompatibility complex
MIP1β: macrophage inflammatory protein 1 beta
P: proline
PBMCs: peripheral blood mononuclear cell
PDB: Protein Data Bank
S: serine
TNF: tumor necrosis factor
Y: tyrosine
PCR: polymerase chain reaction
